# A Randomized, Double-Blind, Sham-Controlled Trial of Transcranial Direct Current Stimulation for the Treatment of Persistent Postural-Perceptual Dizziness (PPPD)

**DOI:** 10.3389/fneur.2022.868976

**Published:** 2022-04-14

**Authors:** Jooyeon Jamie Im, Seunghee Na, Sanghoon Kang, Hyeonseok Jeong, Eek-Sung Lee, Tae-Kyeong Lee, Woo-Young Ahn, Yong-An Chung, In-Uk Song

**Affiliations:** ^1^Department of Psychology, Seoul National University, Seoul, South Korea; ^2^Department of Neurology, Incheon St. Mary's Hospital, The Catholic University of Korea, Seoul, South Korea; ^3^Department of Psychiatry, Yale University, New Haven, CT, United States; ^4^Department of Nuclear Medicine, Incheon St. Mary's Hospital, The Catholic University of Korea, Seoul, South Korea; ^5^Department of Neurology, Soonchunhang University Bucheon Hospital, Bucheon, South Korea

**Keywords:** dizziness, persistent postural-perceptual dizziness, neuromodulation, transcranial direct current stimulation, single photon emission computed tomography

## Abstract

**Background:**

Persistent postural-perceptual dizziness (PPPD) is a functional vestibular disorder that causes chronic dizziness interfering with daily activities. Transcranial direct current stimulation (tDCS) has reportedly improved dizziness in patients with phobic postural vertigo in an open-label trial. However, no randomized, double-blind, sham-controlled study has been conducted on its therapeutic efficacy in PPPD.

**Objective:**

This study was conducted to investigate the efficacy and safety of tDCS as an add-on treatment to pharmacotherapy in patients with PPPD. In addition, functional neuroimaging was used to identify the neural mechanisms underlying the effects of tDCS.

**Materials and Methods:**

In a randomized, double-blind, sham-controlled trial, 24 patients diagnosed with PPPD were randomized to receive active (2 mA, 20 min) or sham tDCS to the left dorsolateral prefrontal cortex (DLPFC), administered in 15 sessions over 3 weeks. The clinical measures that assess the severity of dizziness, depression, and anxiety were collected at baseline, immediate follow-up, 1-month follow-up, and 3-month follow-up. Adverse events were also observed. The effect of tDCS on regional cerebral blood flow (rCBF) was evaluated with single photon emission tomography before and after tDCS sessions.

**Results:**

For the primary outcome measure of the Dizziness Handicap Inventory (DHI) score, a significant main effect of time was found, but neither the treatment-by-time interaction effect nor the main effect of treatment was significant. For the Hamilton Depression Rating Scale (HDRS) score, there was a statistical significance for the treatment-by-time interaction effect and the main effect of time, but not for the main effect of treatment. However, the treatment-by-time interaction effect and the main effect of time on HDRS score appear to be due to one data point, an increase in depressive symptoms reported by the sham group at the 3-month follow-up. For the Activities-specific Balance Confidence (ABC) Scale and the Hamilton Anxiety Rating Scale scores, there were no significant main effects of time, treatment, and treatment-by-time interaction. In a comparison with the changes in rCBF between the groups, a significant treatment-by-time interaction effect was found in the right superior temporal and left hippocampus, controlling for age and sex.

**Conclusion:**

Active tDCS was not found to be significantly more efficacious than sham tDCS on dizziness symptoms in patients with PPPD. It is conceivable that tDCS targeting the DLPFC may not be an optimal treatment option for reducing dizziness symptoms in PPPD. Our findings encourage further investigation on the effects of tDCS in PPPD, which considers different stimulation protocols in terms of stimulation site or the number of sessions.

**Clinical Trial Registration:**

cris.nih.go.kr, identifier: KCT0005068.

## Introduction

Persistent postural-perceptual dizziness (PPPD) is a chronic functional vestibular disorder that is characterized by waxing and waning non-spinning dizziness for more than 3 months (1). PPPD is a common disorder, which comprises about one-fifth of patients who visited the tertiary care dizziness clinic (2, 3). Prior to being established as a new diagnostic term, there were several clinical syndromes of functional dizziness such as chronic subjective dizziness (CSD) (4, 5), phobic postural vertigo (PPV) (6), visual vertigo (VV) (7), and space-motion discomfort (SMD) (8, 9). The unifying diagnosis of PPPD was established incorporating several descriptions of these syndromes by the Bárány Society in 2017, and it is now included in the International Classification of Diseases 11th Revision (ICD-11) (10).

While there are available treatments with some evidence of benefit including pharmacology, vestibular rehabilitation, and cognitive behavioral therapy, there are currently no gold standard guidelines for the treatment of PPPD (11, 12). Regarding pharmacological treatment, selective serotonin reuptake inhibitors (SSRIs) or serotonin norepinephrine reuptake inhibitors (SNRIs) are the first line of treatment options and are most commonly used. It has been reported that SSRI may indirectly interfere with dizziness symptoms by reducing anxiety and depressive symptoms, which are often observed in patients with PPPD (12). Moreover, it remains a possibility that SSRI may directly have effects on the vestibular nuclear complex, which is related to motion-sensitive neural pathways (13). Nonetheless, among patients who properly received SSRI treatment, more than 30% of them reported no significant benefits (14, 15). Therefore, developing another treatment option is a priority.

Transcranial direct current stimulation (tDCS) is a neuromodulation technique that applies a weak current to the scalp for modulating neuronal activity (16). tDCS has been shown to have therapeutic effects in various neurologic and psychiatric disorders (17). An open-label pilot study conducted in patients with PPV, which administers 5 daily sessions of tDCS, has shown to have acute effects on dizziness symptoms (18). However, this trial did not include sham controls and only observed short-term treatment effects.

Furthermore, while neuroimaging studies of PPPD have shown dysfunctional brain activities in regions related to balance and postural control (19), no studies have applied neuroimaging methods to study neural mechanisms associated with treatment effects in PPPD. Hence, the goal of this study was to investigate the therapeutic efficacy of tDCS combined with the medications in patients with PPPD using a double-blind, sham-controlled design. In addition, single photon computed tomography (SPECT) was used before and after the treatment to investigate the changes in regional cerebral blood flow (rCBF) associated with treatment effects.

## Materials and Methods

### Participants

Patients with PPPD between the ages of 18 and 69 years were recruited from the neurology outpatient clinic. The diagnosis of PPPD was made by a licensed neurologist (SN) according to the diagnostic criteria of PPPD, established by the Classification Committee of the Bárány Society (10). All patients were tDCS naive and it was predetermined that patients who receive at least 80% of tDCS treatment (12 out of 15 sessions) were considered adherent. To minimize the possible confounds of side effects, patients were recommended to start and continue maintenance treatment with SSRIs for at least 3 weeks prior to and during the study period. Exclusion criteria were neurological disorders other than PPPD, psychiatric disorders (e.g., generalized anxiety disorder or major depressive disorder), contraindications to tDCS (e.g., metallic implants in the head or history of seizure), history of head trauma with loss of consciousness, and pregnancy. The study was approved by the Institutional Review Board of the Incheon St. Mary's Hospital and was conducted in accordance with the Declaration of Helsinki. The study protocol was registered in the Clinical Research Information Service (Registration Number: KCT0005068).

### Study Protocol

This study was a clinical trial that used a randomized, double-blind, sham-controlled design. Following screening evaluation, patients were randomized to receive active tDCS or sham tDCS. Randomization was performed using a computer-based random number-producing algorithm by a research staff who was not involved in the data collection (HJ). A neurological examination, clinical interview, self-report measures, and brain perfusion SPECT were performed at the baseline and within 1 week after completing the 3-week treatment (immediate follow-up). Additionally, a neurological examination, clinical interview, and self-report measures were collected at 1 and 3 months after the end of treatment. At the baseline, a research staff (JJI) provided training on tDCS self-administration and ensured that participants could independently set up and use the tDCS device, so they can safely perform tDCS at home.

### Transcranial Direct Current Stimulation

Active or sham tDCS was administered for a total of 15 sessions (20 min per session) over 3 weeks *via* two surface electrodes with saline-soaked sponges (6 cm in diameter) using the YDS-301 N device (YBrain Inc, South Korea). The anodal electrode was placed over the left dorsolateral prefrontal cortex (DLPFC) (F3; International 10–20 EEG system) and the cathodal electrode over the right DLPFC (F4). For the active condition, the current was ramped up to 2.0 mA (current density, 0.07 mA/cm^2^) over 30 s, remained at 2.0 mA for 19 min, and ramped down to 0 mA over 30 s. For the sham condition, the current was ramped up to 2 mA over 30 s and ramped down over the next 30 s. The tDCS device was set to be used only one time a day, and the usage logs were automatically stored after each session. Compliance was checked based on the usage logs when the device was returned at the immediate follow-up. During stimulation, patients were instructed not to fall asleep and they were allowed to go about their regular activities. Participants were asked to fill out a survey on side effects after each session. The side effects survey included 13 items, namely burning sensation, dizziness, fatigue, headache, itchiness, nausea, neck pain, pain at the stimulation site, redness of the skin, reduced concentration, sleepiness, tingling sensation, and others. Adequacy of blinding to treatment condition was assessed at the immediate follow-up by asking the participants to guess the treatment condition (active, sham, or do not know) that they were assigned to.

### Clinical Measures

All patients underwent a detailed neurologic examination including neuro-otologic assessment with Frenzel glasses including various positional maneuvers, head-shaking test, bedside head impulse test, and oculomotor tests. Dizziness was rated with self-report measures including the Dizziness Handicap Inventory (DHI), which was the primary outcome measure, and the Activities-specific Balance Confidence (ABC) scale. Handedness was measured using the Edinburgh Handedness Inventory. The severity of depressive and anxiety symptoms was measured with the clinician-administered 17-item Hamilton Depression Rating Scale (HDRS) and the 14-item Hamilton Anxiety Rating Scale (HARS), respectively.

### Brain SPECT Imaging Acquisition and Analysis

The SPECT images were obtained with a dual-headed gamma camera (Discovery NM630; GE Healthcare, Milwaukee, WI, USA) equipped with a low-energy fan-beam collimator. Prior to scanning, patients were injected with 555–740 MBq of technetium-99m ethyl cysteinate dimer (99mTc-ECD) and rested for about 40 min. The images were taken by rotating the camera a total of 720° at 6-degree intervals at a rate of 12 s per frame. Using a filtered back-projection technique, the images were corrected for attenuation and reconstructed into a 128 x 128 matrix with a pixel size of 1.95 mm^3^ × 1.95 mm^3^ × 2.08 mm^3^.

Statistical Parametric Mapping 12 (SPM12; Wellcome Department of Cognitive Neurology, London, UK) implemented in MATLAB R2021 (MathWorks, Natick, MA, USA) was used for preprocessing and analysis. The SPECT images were spatially normalized to the SPM-provided standard SPECT template, which is based on the Montreal Neurological Institute (MNI) template. After spatial normalization, each voxel count was standardized to the mean voxel count of the whole brain using proportional scaling. The normalized SPECT images were then smoothed with a 16-mm full-width half-maximum Gaussian kernel.

Voxel-wise comparisons were performed using (1) a two-sample *t*-test model to investigate rCBF difference between groups at baseline, (2) a flexible factorial model to identify treatment (anode and sham) × time (baseline and immediate follow-up) interaction effects on rCBF, and (3) as a *post hoc* analysis, a paired *t*-test model to investigate rCBF changes within each group before and after the treatment. Statistical significance level was defined as a *p*-value threshold of 0.001 and a minimum cluster size of 50 contiguous voxels.

### Statistical Analysis

Baseline characteristics of groups were compared using independent *t*-test for normally distributed continuous variables, Wilcoxon rank-sum test for non-normally distributed continuous variables, and chi-square test for categorical variables. A mixed-effects linear regression model was conducted to assess the interaction effect of treatment (anode and sham) and time (baseline, immediate, 1-month, and 3-month follow-ups) for each outcome measure. To better understand the interaction effects between group and time, a two-sample *t*-test was performed between active and sham groups at each visit. Reported side effects were compared between groups using the chi-square test. All statistical analyses were performed using R software (version 4.0.5).

## Results

### Participants

**Figure 1:**
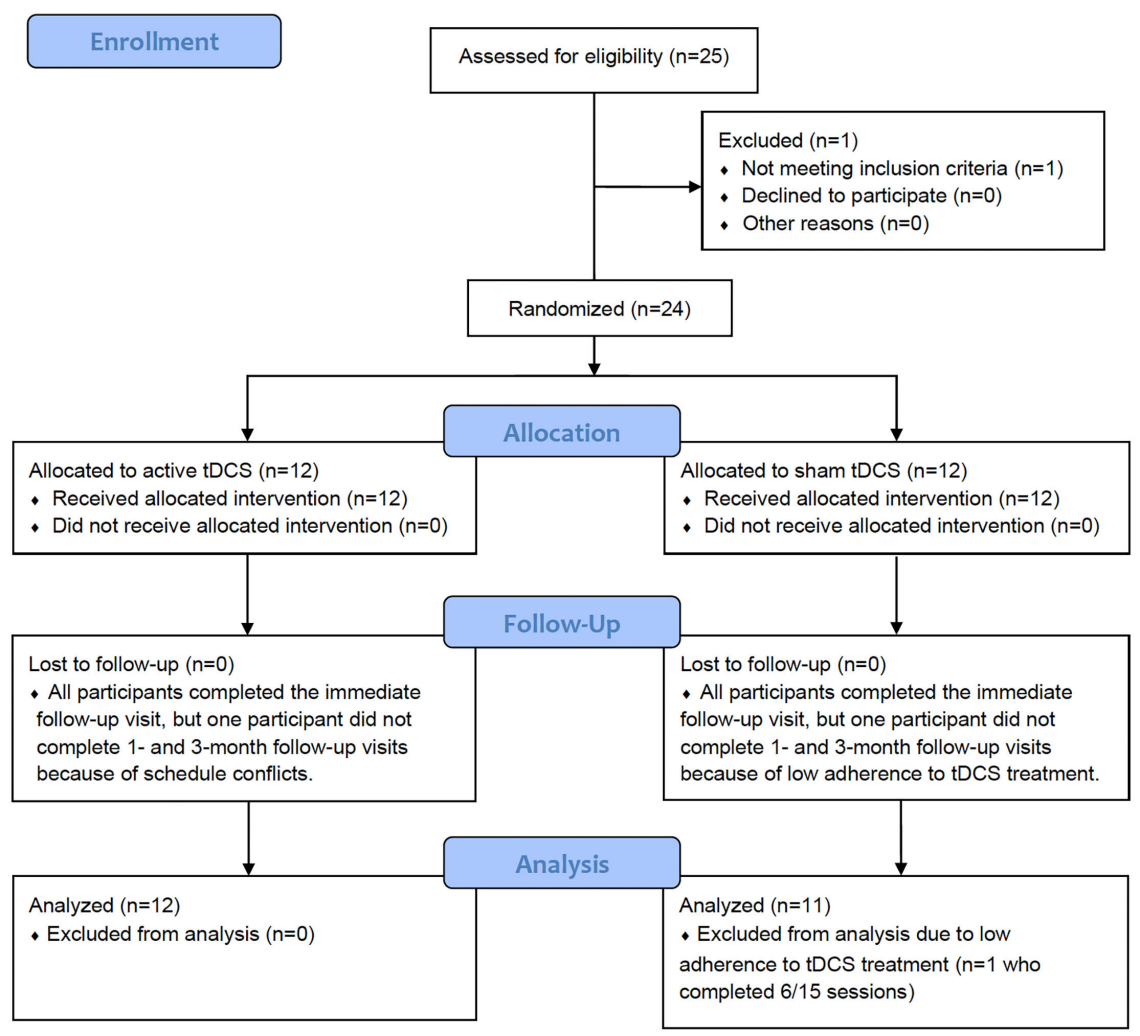
Flow diagram for study participants.

Among 24 participants who were randomized into active (*n* = 12) or sham (*n* = 12) groups, 23 participants were included in the final analysis after excluding one participant in the sham group due to low adherence to tDCS treatment (Figure 1). The baseline demographic and clinical characteristics of the participants did not differ between active and sham groups (Table 1).

**Table 1:** Baseline demographic and clinical characteristics of the study participants.

**Characteristic**	**Active group** **(*n* = 12)**	**Sham group** **(*n* = 11)**	***p*** **-value**
Age (years)	47.8 (13.0)	51.7 (13.1)	0.48
Sex (male:female)	4:8	4:7	1.00
Education (years)	11.6 (3.9)	11.9 (3.1)	0.90
Disease duration (months)	17.6 (396.3)	14.8 (348.0)	0.65
Medication	10 (8 with Escitalopram, 2 with Hypericum extracts), 2 with no medication	9 (8 with Escitalopram, 1 with Sertraline), 2 with no medication	0.99
DHI score	34.3 (15.9)	35.3 (14.2)	0.88
ABC score	77.3 (21.0)	77.6 (17.5)	0.90
HDRS score	5.4 (3.2)	5.8 (5.6)	0.81
HARS score	6.6 (3.2)	7.6 (6.4)	0.63

*Data are presented as mean (standard deviation) or numbers*.

*ABC, activities-specific balance confidence; DHI, dizziness handicap inventory; HARS, hamilton anxiety rating scale; HDRS, hamilton depression rating scale*.

Neurologic examinations at the baseline and follow-ups revealed no abnormal findings except for subtle positional nystagmus in 1 patient and transient head-shaking nystagmus in 4 patients. Precipitating events of PPPD were reported as benign paroxysmal postural vertigo (*n* = 8), vestibular neuritis (*n* = 5), other systemic diseases such as flu (*n* = 1) or cardiovascular symptoms (*n* = 3), emotional stress (*n* = 3), mild head trauma without loss of consciousness (*n* = 1), and none (*n* = 2).

Prior to study entry, all participants started taking either SSRI [20 participants with escitalopram (8.82 ± 4.52 mg per day) and 1 with sertraline (25 mg per day)] or herbal medicine (2 participants with hypericum extracts known for antidepressant effects). Then, four out of 20 participants taking escitalopram discontinued the medication due to the side effects (*n* = 3 for gastrointestinal effects and *n* = 1 for loss of libido), and all other participants took their respective medication during tDCS treatment and the dosage did not change during the trial.

Regarding the validity of the blinding procedure, most participants from both groups (21/23) either guessed that the treatment they received was active treatment or was unsure. In the active group, 6 participants (50%) answered that they received active treatment (correctly guessing their treatment group) and 6 participants (50%) answered, “don't know.” In the sham group, 7 participants (63.63%) answered that they received active treatment (incorrectly guessing their treatment group), 2 participants (18.18%) answered that they received sham treatment (correctly guessing their treatment group), and 2 participants (18.18%) answered, “don't know.” Two participants (*n* = 1 in active and n = 1 in sham groups) were left-handed, and the rest were right-handed. The number of participants who experienced the side effects during tDCS treatment for each group is reported in **Table 4**.

### Clinical Outcomes

**Figure 2:**
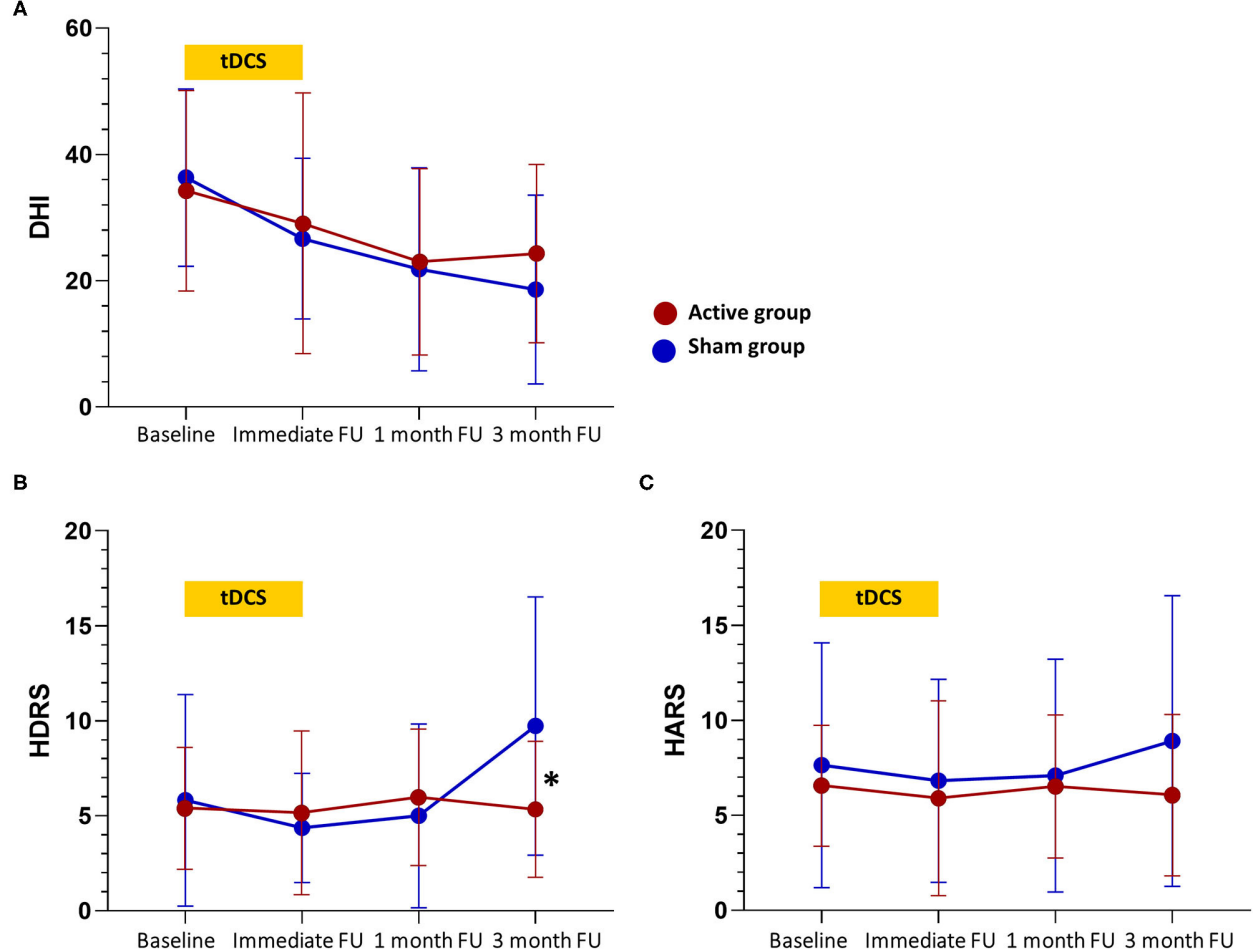
Changes in clinical scores over time. **(A)** Changes in the Dizziness Handicap Inventory (DHI) total score. **(B)** Changes in the Hamilton Depression Rating Scale (HDRS) score. **(C)** Changes in the Hamilton Anxiety Rating Scale (HARS) score. tDCS sessions were between baseline and immediate follow-up. Values are mean scores and error bars indicate 95% confidence intervals. Significant differences at the *p* < 0.05 level between groups at a time point are denoted with a “*”. FU, follow-up.

Results for the clinical outcomes are illustrated in Table 2 and Figure 2. For the primary outcome measure of DHI score, a significant main effect of time was found (*p* < 0.001), but neither the main effect of treatment (*p* = 0.79) nor the treatment-by-time interaction effect (*p* = 0.58) was significant. *Post hoc* analysis revealed no significant group difference in the DHI score at each time point. For the ABC score, there was no significant main effect of time (*p* = 0.44), treatment (*p* = 0.45), and treatment-by-time interaction effect (*p* = 0.35).

**Table 2:** Summary of clinical variables across time and group.

**Measures**	**Anode**	**Sham**	**Treatment** **(active vs. sham)**		**Time**		**Treatment x Time**	
			***F***	***p*** **-value**	***F***	***p*** **-value**	***F***	***p*** **-value**
**DHI**								
Baseline	34.33 (15.9)	35.27 (14.2)	0.07	0.79	14.79	<0.001	0.66	0.58
Immediate F/U	29.17 (20.6)	25.64 (12.8)						
1-month F/U	23.1 (14.7)	21.8 (16.1)						
3-month F/U	25.1 (13.6)	19.1 (14.3)						
**ABC**								
Baseline	77.34 (21.0)	77.61 (17.5)	0.59	0.45	0.91	0.44	1.10	0.35
Immediate F/U	72.03 (26.6)	81.82 (15.9)						
1-month F/U	75.57 (21.9)	82.84 (16.4)						
3-month F/U	75.34 (24.7)	85.00 (15.1)						
**HDRS**								
Baseline	5.42 (3.20)	5.82 (5.56)	0.29	0.60	3.75	0.02	4.65	0.005
Immediate F/U	5.17 (4.30)	4.36 (2.87)						
1-month F/U	6.0 (3.58)	5.0 (4.84)						
3-month F/U	5.36 (3.59)	9.73 (6.80)						
**HARS**								
Baseline	6.58 (3.18)	7.64 (6.44)	0.54	0.47	0.48	0.70	0.66	0.58
Immediate F/U	5.92 (5.11)	6.82 (5.34)						
1-month F/U	6.55 (3.75)	7.09 (6.12)						
3-month F/U	6.09 (4.23)	8.91 (7.65)						

*Data are presented as mean (standard deviation). Statistical test results are from linear mixed-effects models with each clinical variable as dependent variables and time and treatment (active vs. sham) as the predictors*.

*Treatment, main effect of treatment; Time, main effect of time; Treatment X Time, interaction effect of treatment and time*.

*ABC, activities-specific balance confidence; DHI, dizziness handicap inventory; F/U, follow-up; HARS, hamilton anxiety rating scale; HDRS, hamilton depression rating scale*.

For the HDRS score, the treatment-by-time interaction effect (*p* = 0.005) and the main effect of time (*p* = 0.02) were significant, but the main effect of treatment (*p* = 0.60) was not significant. *Post hoc* analysis showed that the HDRS score was lower in the active group compared to the sham group at the 3-month follow-up (5.36 vs. 9.73, *p* = 0.02). Notably, the treatment-by-time interaction effect and the main effect of time on HDRS score appear to be due to an increase in depressive symptoms reported by the sham group at the 3-month follow-up. Considering that the minimal clinically important difference (MCID) is 4–6 points for the HDRS-17, there was no clinically meaningful difference in the HDRS score within or between groups at any other time point (20). For the HARS score, there were no significant main effect of time (*p* = 0.70), main effect of treatment (*p* = 0.47), and treatment-by-time interaction effect (*p* = 0.58).

### Brain SPECT Imaging

**Figure 3:**
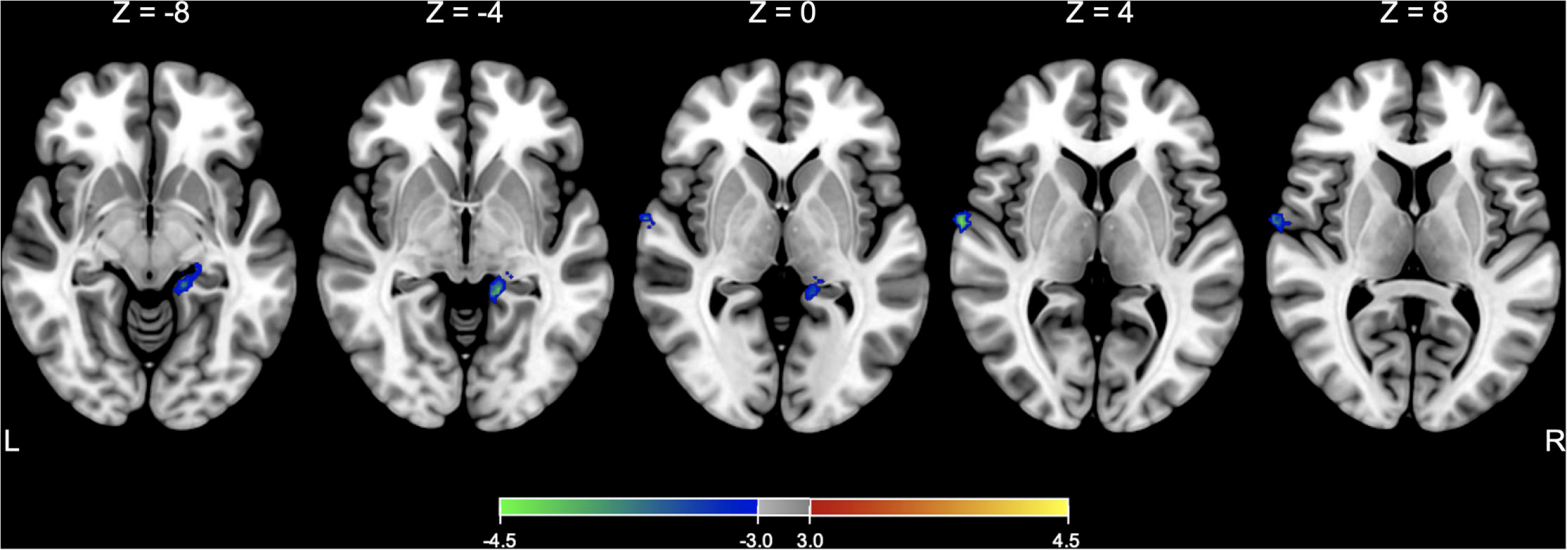
SPECT analysis results. Significant areas with treatment (active vs. sham) x time (baseline vs. immediate follow-up) interaction effects of rCBF are overlaid on the Montreal Neurological Institute (MNI) 152 template. Color bar represents the voxel-level *t*-values.

Before testing models of interest, a two-sample *t*-test model was conducted to compare the baseline SPECT images between the active and sham groups and found that there was no significant difference in rCBF between groups at the baseline. In comparison with the changes in rCBF between the groups, a flexible factorial model identified a significant treatment-by-time interaction effect in the right superior temporal gyrus and left hippocampus or parahippocampus, controlling for age and sex (Table 3 and Figure 3). *Post hoc* analysis of a paired *t*-test showed that the rCBF from the left superior frontal gyrus and left hippocampus was significantly decreased in the active group between the baseline and immediate follow-up, but no changes were observed in the sham group (Table 3).

**Table 3:** Information on the significant clusters for both the interaction contrast and the within-group contrast.

**Region**	***t***	***p***	**Coordinates** **(x, y, z)**	**Cluster size (voxels)**
**Interaction**				
***Anode** **<** **sham***				
Right superior temporal gyrus	4.78	<0.001	66, −2, 2	75
Left hippocampus	4.3	<0.001	−20, −32, −10	127
**Within-Anode**				
***Baseline** **>** **immediate follow-up***				
Left hippocampus	6.55	<0.001	−20, −32, 4	61
Left superior frontal gyrus	6.14	<0.001	−24, 54, 10	95
**Within-Sham**				
No significant findings

*All analyses used models with age and sex as covariates. The coordinates refer to the Montreal Neurological Institute coordinate system*.

### Safety Outcomes

All participants filled out a survey on side effects after each session. The number of participants who reported side effects is summarized by groups in Table 4. The most reported side effects were transient dizziness and itchiness in both active and sham groups. No significant group differences were observed for all side effects.

**Table 4:** Reported side effects between treatment groups.

**Side effects**	**Active group** (*n* = 11)^*^	**Sham group** **(*n* = 11)**	***p*** **-value**
Dizziness (*n*)	6	6	1.00
Itchiness (*n*)	6	4	0.81
Headache (*n*)	5	3	0.66
Tingling sensation (*n*)	5	3	0.66
Pain at the stimulation site (*n*)	3	3	1.00
Sleepiness (*n*)	3	2	1.00
Neck pain (*n*)	3	1	0.58
Redness of skin (*n*)	3	1	0.58
Reduced concentration (*n*)	3	1	0.58
Fatigue (*n*)	3	2	1.00
Nausea (*n*)	1	2	1.00
Burning sensation (*n*)	1	2	1.00

* *Side effects records for 1 participant from the active group were missing*.

## Discussion

To the best of our knowledge, this was the first randomized, double-blind, sham-controlled trial that investigated the effects of tDCS in patients with PPPD. Our study found no significant beneficial effects of tDCS on dizziness symptoms and balance confidence, measured by DHI and ABC scale, respectively. Regarding psychiatric symptoms, there was no clinically meaningful effect of anodal tDCS on depressive and anxiety symptoms measured by HDRS and HARS, respectively.

The demographic characteristics of the participants were typical of PPPD. Mean age was middle-aged and female predominance was noted. Most patients reported that precipitating events were peripheral vestibular disorder but two patients could not identify any specific trigger. The failure to identify precipitants could be attributed in part to the patients regarding the triggers as trivial or having difficulty linking the seemingly unrelated triggers to the development of their symptoms.

The absence of tDCS effects on dizziness symptoms in PPPD patients contradicts the previous open-label study, which reported a significant reduction of DHI scores on the last day of the 5-day tDCS treatment in patients with PPV; however, these effects were transient and were not observed during the 1, 2, and 4-week follow-ups (18). In addition, although not statistically significant, this previous study showed a tendency for decreased depression and anxiety symptoms after the treatment compared to the baseline. Due to the absence of a control group and the small sample size, the results from this previous study should be interpreted with caution. For the DHI, our study revealed that there was a significant main effect of time. The gradually decreased dizziness symptoms with time in both groups could be due to the effects of SSRIs, as the treatment efficacy of serotonergic medication in PPPD has been proven in the previous studies (4, 14, 21).

Unexpectedly in our study, the severity of depressive symptoms increased at the 3-month follow-up in the patients of the sham group, whereas the severity of depressive symptoms remained stable in the patients of the active group. These results were not due to the outliers as the baseline HDRS scores were increased at the 3-month follow-up in 6 patients in the sham group, which is almost half of the patients in the group. The reasons for this isolated increase in the HDRS score in the sham group at the 3-month follow-up are unclear but may be due to the clinical outcome not meeting patients' expectations, frustration or anger with sustained dizziness symptoms, or initially underestimated baseline symptom (17, 22).

In our study, the brain SPECT analysis revealed a significant treatment-by-time effect on rCBF in the left hippocampus and right superior temporal areas. The *post hoc* analysis showed that the rCBF was decreased at the immediate follow-up compared to the baseline only in the active group. Considering that these regions are regarded as the components of multimodal vestibular cortical areas (22), this tDCS protocol might have unintentionally exacerbated the maladapted brain function to the extent that clinical symptoms did not worsen.

Several assumptions could be made concerning the absence of tDCS effects in patients with PPPD. First, it is possible that the target area for stimulation might be inappropriate. The left DLPFC was chosen as the target area based on the previous tDCS studies in PPV (18) and also in various neurological and psychiatric disorders including major depression disorder, addiction or craving, tinnitus, and neurodegenerative disorders (23). We expected that anodal tDCS would have positive effects on chronic dizziness by ameliorating various cognitive functions related to the high-risk postural control strategies, stimulating the functionally decreased prefrontal regions (24), and improving possibly coexisting psychiatric symptoms such as depressive or anxious mood in patients with PPPD. Contrary to the expectations, anodal tDCS did not improve dizziness symptoms compared to sham tDCS. In functional neuroimaging studies in PPPD, it was reported that the local activity and connectivity in the multimodal vestibular area are usually decreased and the connectivity between the prefrontal and primary visual areas is usually increased, which is related to the over-reliance on the visual stimuli (19, 24). In neurological disorders with chronic pain, the M1 (the primary motor cortex) area is adopted as the target area other than the dorsolateral prefrontal region (25). Patients with fibromyalgia and diabetic polyneuropathy who received tDCS stimulation on the M1 area showed efficacy on various pain measures but studies targeting the DLPFC were failed to show such effects (25, 26). Like these cases, changing the stimulation target might have therapeutic effects and should be tested in future studies. For example, tDCS stimulation on the multimodal vestibular cortical areas could enhance the local activity and connectivities to other relevant regions and may directly induce the improvement of dizziness symptoms.

The lack of tDCS efficacy may also be due to the suboptimal parameters of the tDCS protocol for PPPD. Whereas the tDCS protocol used in this study consists of 15 tDCS sessions, the pilot study of tDCS on PPV was conducted with only 5 sessions (18). In the previous studies of major depressive disorder and fibromyalgia, about 10 sessions were commonly conducted (25, 27). On the other hand, a much greater number of sessions with longer periods have been used in several previous studies (28, 29). In addition, other treatment modalities of PPPD such as vestibular rehabilitation or medications are needed up to 12 weeks to achieve sustained benefit. Thus, the number of sessions in this study might be inappropriate. Finally, the interaction with medication also could be another reason for the lack of tDCS effects (30, 31).

This study has strengths in that, in spite of a relatively small sample size, it utilized a larger sample size than the only existing study regarding the effects of tDCS on PPV (18) and is the first double-blind study with a sham-controlled condition. Moreover, this study demonstrated the feasibility of home-based treatment, of which its importance is being acknowledged especially in this pandemic era. Considering the reported adverse events in our study and the dropout rate of the enrolled patients, the home-based tDCS appears to be safe and easy to apply. This study has several limitations. We did not perform any prior power analysis. Therefore, due to the underpowered design, it cannot be ruled out that we may not detect the true effect of tDCS. Moreover, in spite of possible drug-induced effects over the tDCS effects, our study was not a monotherapy trial as it was difficult to conduct a study that would restrict patients from receiving SSRI/SNRIs, medications in which early studies have shown some benefits for treating PPPD, for more than 3 months in a clinical setting.

In conclusion, active tDCS targeting the left DLPFC was not found to be significantly more efficacious than sham tDCS on dizziness symptoms in patients with PPPD. Considering the high incidence of PPPD and the overall response rate of serotonergic medication, developing an alternative treatment option is needed. Our findings encourage further investigation on the effects of tDCS in PPPD, which considers different stimulation protocols in terms of stimulation site or the number of sessions.

## Data Availability Statement

The raw data supporting the conclusions of this article will be made available by the authors, without undue reservation.

## Ethics Statement

The studies involving human participants were reviewed and approved by the Institutional Review Board of the Incheon St. Mary's Hospital. The patients/participants provided their written informed consent to participate in this study. Written informed consent was obtained from the individual(s) for the publication of any potentially identifiable images or data included in this article.

## Author Contributions

JI, SN, Y-AC, and I-US: conceived and designed the study. JI and Y-AC: obtained the funding. Y-AC, I-US, T-KL, and W-YA: provided supervision. JI, E-SL, and SN: collected the data. HJ: independently performed the randomization process. JI, SK, and SN: performed data analysis and wrote the first draft of the manuscript. All authors contributed to manuscript revisions and read and approved the final version of the manuscript for submission.

## Funding

This research was supported by the Basic Science Research Program through the National Research Foundation of Korea (NRF) and funded by the Ministry of Science and ICT (2018R1A6A3A11041118).

## Conflict of Interest

The authors declare that the research was conducted in the absence of any commercial or financial relationships that could be construed as a potential conflict of interest.

## Publisher's Note

All claims expressed in this article are solely those of the authors and do not necessarily represent those of their affiliated organizations, or those of the publisher, the editors and the reviewers. Any product that may be evaluated in this article, or claim that may be made by its manufacturer, is not guaranteed or endorsed by the publisher.
